# Initial stability of one-stage anterior debridement and cage implantation combined with anterior-lateral fixation by a dual screw-rod construct in the treatment of lumbosacral tuberculosis: a cadaveric biomechanical study

**DOI:** 10.1186/s12891-019-2592-2

**Published:** 2019-05-10

**Authors:** Jiantao Liu, Yanzheng Gao, Zhengchao Gao, Binbin Niu, Dongbo Lv, Yin Yang

**Affiliations:** 1 Department of Spine and Spinal Cord Surgery, Henan Provincial People’s Hospital, Zhengzhou, Henan Province China; 2 Department of Orthopedics, Second Affiliated Hospital of Xi’an Jiaotong University, Xi’an, Shaanxi Province China; 3 Department of Orthopedics, Xi’an Central Hospital, Xi’an, Shaanxi Province China

**Keywords:** Lumbosacral tuberculosis, Subtotal resection, Anterior approach, Titanium cage, Biomechanics

## Abstract

**Background:**

Although various surgical methods are used to treat lumbosacral tuberculosis, no unified surgical approach exists. Thus, exploring an optimal operation method has substantial clinical importance. Evaluate the initial stability of a new surgical method, a one-stage anterior debridement and cage implantation combined with anterior-lateral fixation by a dual screw-rod construct, in the treatment of lumbosacral tuberculosis and provide biomechanical support for its further promotion in clinical applications.

**Methods:**

Fifteen fresh human lumbosacral spine specimens without fractures, deformities or osteoporosis were randomly divided into intact (I), anterior fixation (AF) and posterior fixation (PF) groups. All AF and PF group specimens had subtotal resections of the L5 vertebra and adjacent discs, while the I group specimens were kept intact. Then, titanium cages were implanted in the surgical site and a dual screw-rod construct was fixed anterior-laterally in the AF group, while the PF group specimens were fixed posteriorly with only the dual screw-rod construct. Mechanical tests were conducted for initial stability evaluations.

**Results:**

The load at the maximum displacement (5 mm) or rotation angle (5 °) was less for the I group specimens than for the AF and PF group specimens in all directions (*P* < 0.05). The load at the maximum displacement (5 mm) was greater for the AF group specimens than for the PF group specimens in flexion, lateral bending and axial compression (*P* < 0.05) and lower than in the PF group specimens in extension (*P* < 0.05). In torsion, there was no difference between the loads in the AF and PF groups at the maximum rotation angle (5 °) (*P* > 0.05).

Conclusions: The proposed surgical approach can provide better immediate stability than anterior debridement with posterior dual screw-rod fixation in the treatment of lumbosacral tuberculosis in flexion, lateral bending and axial compression.

## Background

Although lumbosacral spinal tuberculosis accounts for only 2–3% of spinal tuberculosis diagnoses [1], lumbosacral spinal tuberculosis often leads to anterior vertebral column destruction and presacral or iliopsoas abscesses [2], which always requires surgery for a permanent cure [2, 3]. Due to the complexity of the anatomical structure of the lumbosacral spine and the high requirement of fixation stability, both anterior and posterior approaches have been applied for the treatment of lumbosacral spinal tuberculosis [4–6]. Although satisfactory results of different approaches have been reported by various authors [7–9], there is no consensus on the optimal approach. It is difficult to achieve thorough debridement by a single posterior approach [10], while single- or two-stage combined anteroposterior approaches create severe trauma and are expensive [11]. A single anterior approach can clear infected lesions under direct vision, but the stability of internal fixation in this approach is controversial. Although anterior lumbosacral internal fixation systems are designed to improve the internal fixation strength [12], these fixation systems cannot be applied to spinal tuberculosis patients who have irregular bony destruction. To remedy the above deficiencies, we developed a new surgical procedure, one-stage anterior debridement and cage implantation combined with anterior-lateral fixation by a dual screw-rod construct underneath the iliac vessel, and obtained satisfactory results according to follow-up investigations [13, 14]. However, some surgeons question the stability of this procedure in the reconstruction of the lumbosacral spine. Therefore, we conducted an in vitro biomechanical study with human specimens to assuage these doubts and lay the foundation for the clinical application of this new method. The reestablished stability of the lumbosacral spine in flexion and extension, lateral flexion, torsion and axial compression was tested by comparing with the traditional anterior lesion removal combined with posterior double screw-rods fixation.

## Methods

### Specimens

The Anatomy and Pathology Department of the Medical College of Xi’an Jiaotong University provided 15 human lumbosacral spine specimens (L3-S3) for research at no cost. The specimens were from 7 males and 8 females, with an average age of 50.4 years. Computed tomography (CT) scanning (0.625 mm, GE Medical Systems, Milwaukee, WI) and bone mineral density (BMD) examinations (MEDIX90, French, Mr. Stowe, France) were performed to exclude specimens with skeletal abnormalities. All remaining specimens were randomly divided into three groups: the intact group (I group), anterior fixation group (AF group) and posterior fixation group (PF group). The muscles and blood vessels of all specimens were carefully removed by an experienced orthopedic surgeon, keeping the ligaments, intervertebral discs, and capsule of the facet joint intact. Then, all the specimens were double-wrapped in plastic bags and stored in a − 20 °C freezer.

### Model building

The frozen specimens were thawed at room temperature for 8 h to unfreeze. A subtotal corpectomy of the L5 vertebra for all specimens in the AF and PF groups was performed by an experienced orthopedic surgeon using a bone rongeur for decompression. The adjacent discs (L4–5 and L5-S1) were removed using a pituitary rongeur. No operations were performed for the specimens in the I group. For the specimens in the AF group, a titanium cage filled with autologous bone was first placed in the surgical site, and then four pedicle screws were inserted anterior-laterally into the L4 and S1 vertebrae. Finally, prebent two titanium rods were connected with the pedicle screws underneath the iliac vessel. For the specimens in the PF group, four pedicle screws were first implanted along the L4 and S1 vertebral arch, and then two prebent titanium rods were connected with the pedicle screws. Some radiological examinations (anterior-posterior and lateral X-ray imaging, CT scanning and 3-D reconstruction) were performed to inspect the prostheses position and the status of spinal cord.

### Biomechanical tests

The biomechanical tests were performed at the State Key Laboratory of Mechanical Strength and Vibration of Xi’an Jiaotong University using An MTS 858 Mini Bionix II biomaterial testing system (MTS, USA). According to the testing criteria reported by H.J. Wilke and coworkers [15], using a special metal mold containing polymethylmethacrylate to embed the both ends of the L4-S3 segments of the specimens, with the lumbosacral segment perpendicular to the horizontal plane.

**Fig. 1:**
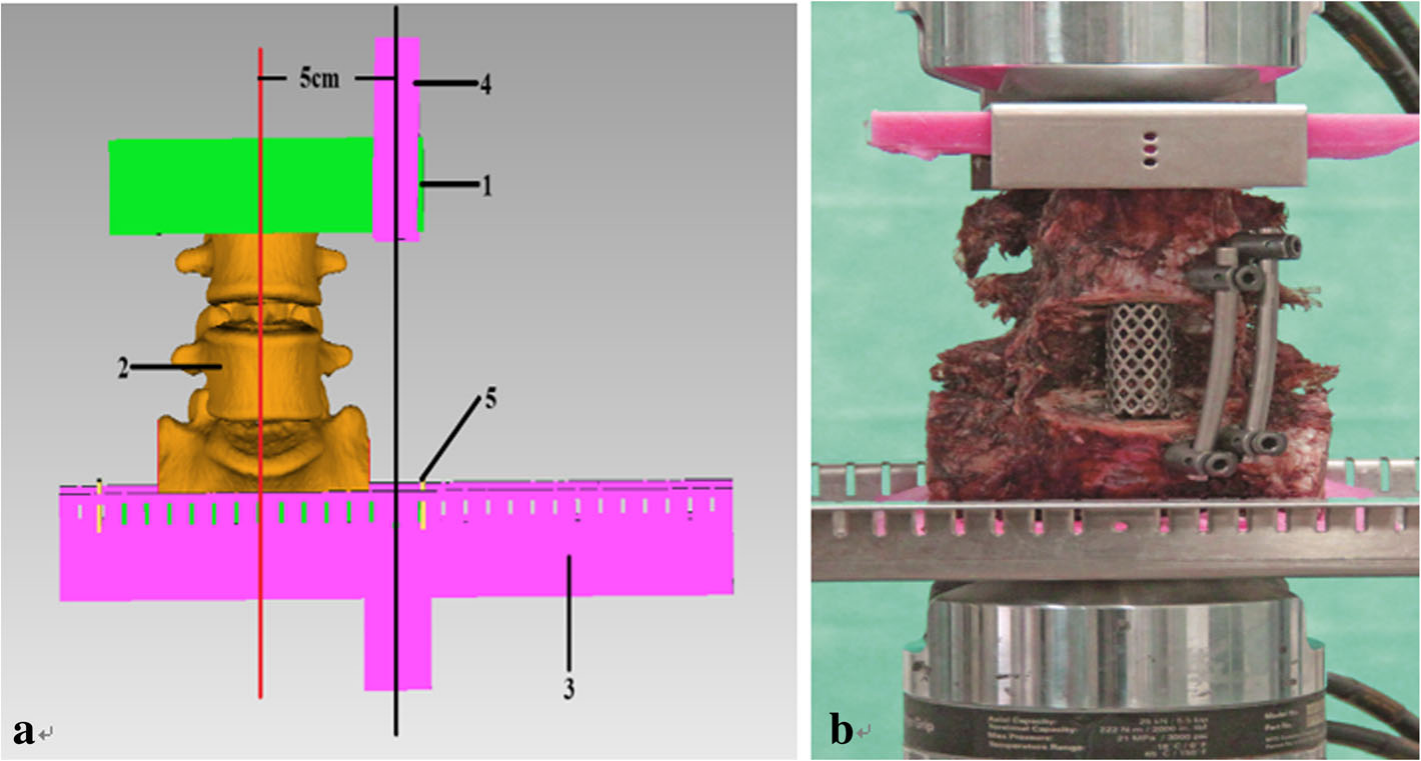
Images of the biomechanical tests: **a** pattern graph of left lateral bending: 1 embedding block, 2 lumbosacral spine, 3 fixed base, 4 narrow load heads, 5 stable films; **b** Images of the AF group in the torsion test

To evaluate the stability of the new surgical method, we conducted bending tests, torsion tests and axial compression tests on the three groups of specimens. The bending test mainly assesses the stabilities in the flexion, extension, and left and right lateral bending directions. Because the MTS 858 Mini Bionix II machine in our laboratory were lack of multidirectional payload head, bending tests cannot be performed directly on specimens. To solve the above problem, we developed a new fixture (Fig. 1a), which could allow the specimens to move to one side. Then, the load could be conducted through the fixture to bend the specimen. The distance between the loading arm and the bottom-center of the specimen was fixed at 5 cm. The specimen was displaced at a rate of 1 mm/min, and the termination condition was set to a displacement of 5 mm, and the displacement-load curve was recorded. The torsion test mainly evaluates the mechanical characteristics in the torsional direction. As the machine could rotate the specimens directly, a specimen was placed in the center of the loading arm of the machine and the load was applied (Fig. 1b). The torsional load on the specimen was applied at a rate of 1 °/min, and the termination angle was set to 5 ° in accordance with a previous study [16]. The rotation angle-load curve was recorded. The axial compression test mainly tests the mechanical properties of axial compression. The specimens were axially compressed at a rate of 1 mm/min, and the maximum loading displacement was set to 5 mm. Each pressure was loaded and released three times in order to minimize the viscoelastic effects, the results of the third load cycle was evaluated and used for further analysis. During the test, spray the specimen with saline every 5 min to keep them moist.

### Statistical analysis

SPSS software (Version 21, International Business Machines Corp., USA) was used to analyze the data. Mean ± S.D. was used to present the results. The loads were analyzed using a one-way analysis of variance (ANOVA). Pairwise comparisons were conducted using the least significant difference (LSD) test. *P* < 0.05 was considered statistically significant.

## Results

### Specimen selection

**Fig. 2:**
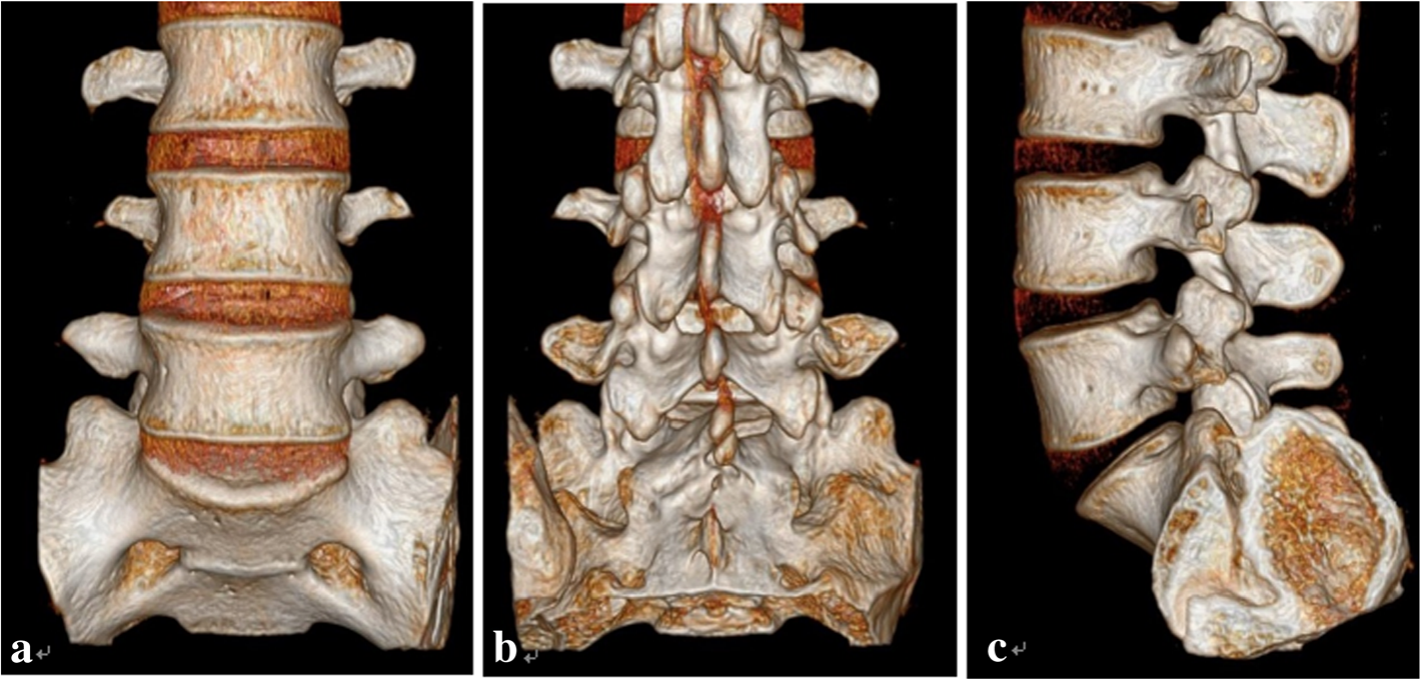
Three-dimensional reconstruction of a lumbosacral vertebral specimen by thin-layer CT scans: **a** front view, **b** dorsal view, and **c** lateral view

Preoperative imaging examination of all specimens showed no fractures, infections, deformities or other spinal diseases (Fig. 2). The BMD examination results showed that the BMDs of all specimens were normal: the average BMD was (1.07 + 0.11) g/cm^2^. No bone loss or osteoporosis was observed in any of the specimens.

### Postoperative imaging

**Fig. 3:**
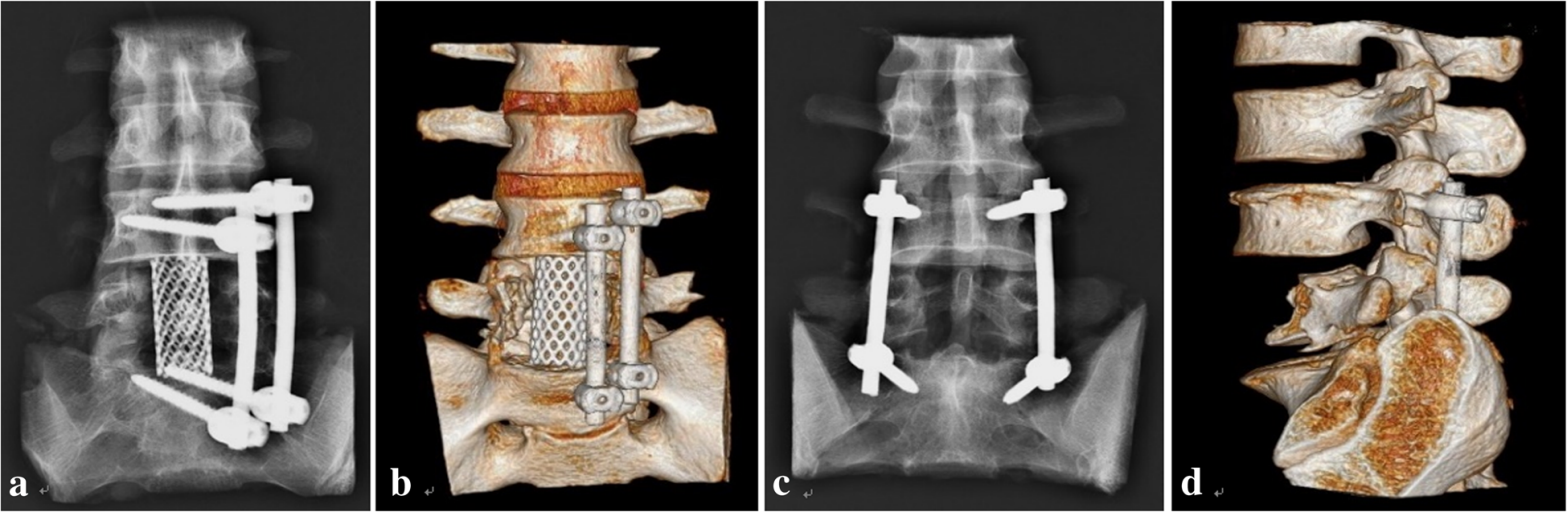
Postoperative imaging examinations of the lumbosacral spine specimens: **a**, **b** the anterior fixation group and **c**, **d** the posterior fixation group

As shown in Fig. 3, all the implants of the lumbosacral spine specimens in the AF and PF groups were appropriately located, and no adverse phenomena, such as pedicle blasts, pedicle screw punctures into the spinal canal or spinal cord compression, were found.

### Test of stability

**Fig. 4:**
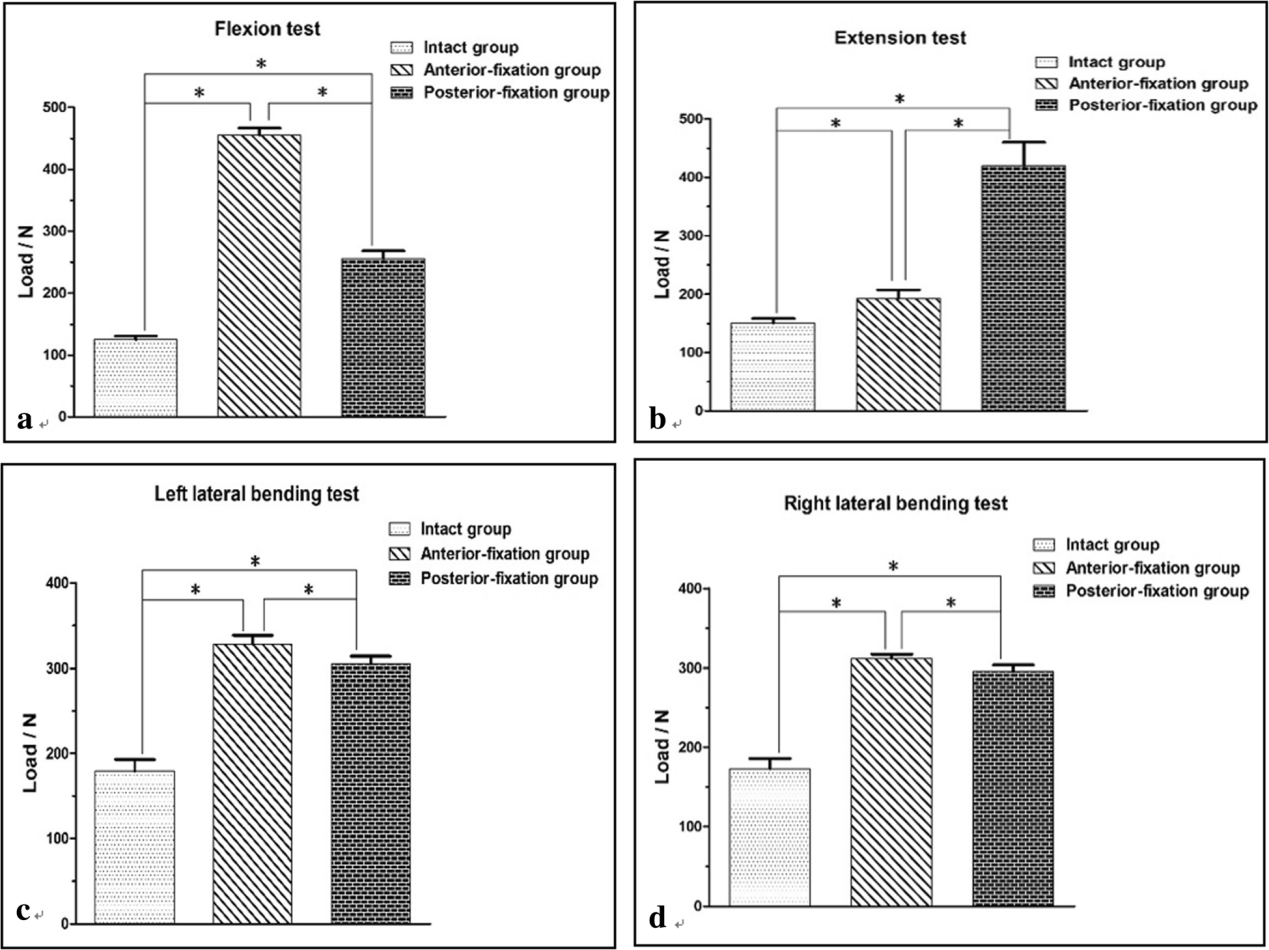
Load comparisons of the specimens in the three groups when the maximum loading displacement was achieved during the bending test: **a** flexion test, **b** extension test, **c** left lateral bending test, and **d** right lateral bending test. *P < 0.05 indicates that the difference was statistically significant

Table 1 and Fig. 4 show the mechanical test results of the three groups in the flexion, extension, and left and right bending directions. The load required to reach the maximum displacement (5 mm) for the specimens in the I group was significantly smaller than that for the specimens in the AF and PF groups (*P* < 0.05). The load required to reach the maximum displacement for the specimens in the AF group was significantly greater than that for the specimens in the PF group in the flexion, left and right lateral bending directions (*P* < 0.05), while in the extension direction, the load required for the specimens in the AF group was significantly smaller than that for the specimens in the PF group (*P* < 0.05).

**Table 1:** Load required for the maximum loading displacement for the specimens in the three groups during the bending test (mean ± S.D., N)

Item	I group	AF group	PF group	t_I-AF_	t_I-PF_	t_AF-PF_
FLX	124.7 ± 5.8	455.3 ± 11.2	255.4 ± 12.3	< 0.001	< 0.001	< 0.001
EXT	150.4 ± 8.1	192.8 ± 14.6	419.7 ± 40.1	0.020	< 0.001	< 0.001
LLB	179.5 ± 13.5	328.2 ± 10.3	304.8 ± 9.1	< 0.001	< 0.001	0.006
RLB	173.1 ± 12.9	311.8 ± 5.2	295.5 ± 7.9	< 0.001	< 0.001	0.016

*FLX* flexion, *EXT* extension, *LLB* left lateral bending, and *RLB* right lateral bending

**Fig. 5:**
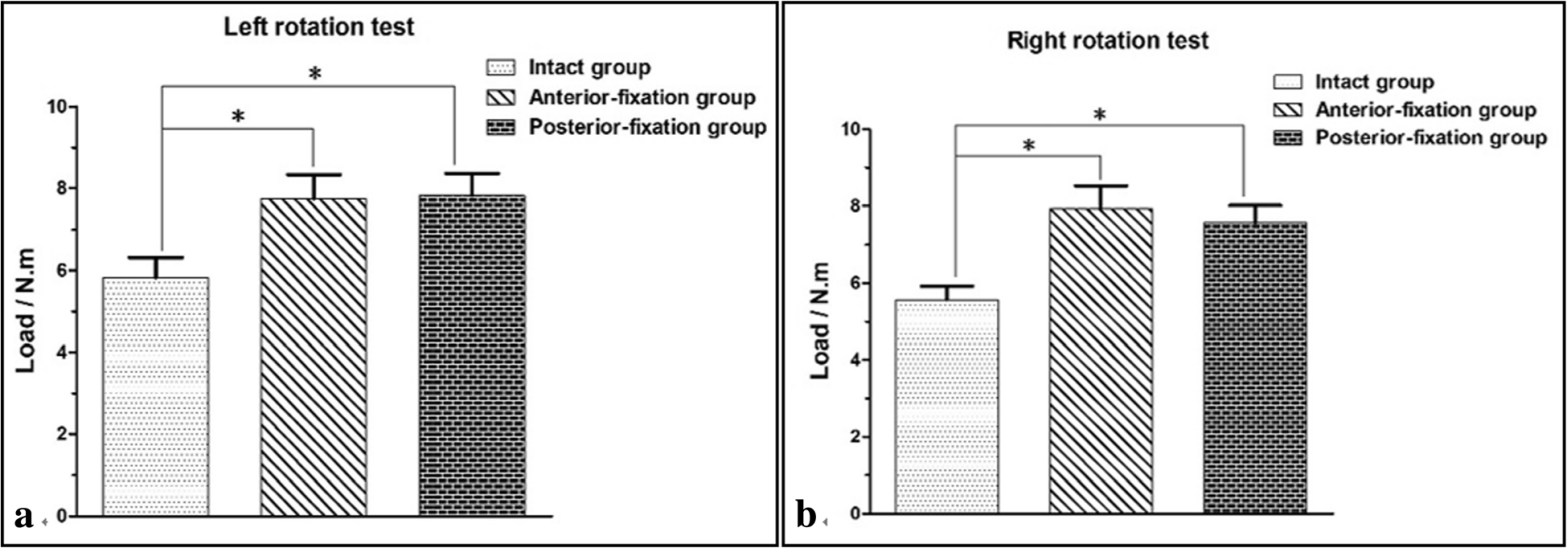
Load comparisons of the specimens in three groups when the maximum rotation angle was reached in the rotation direction: **a** left rotation test and **b** right rotation test. *P < 0.05 indicates that the difference was statistically significant

When reaching the maximum rotation angle (5 °), the specimens in the I group required a smaller load than those in the AF and PF groups in the left-right rotation direction (*P* < 0.05). However, there was no difference in load between the specimens in the AF and PF groups (*P* > 0.05). The detailed results are shown in Table 2 and Fig. 5.

**Table 2:** Load required to reach the maximum rotation angle for the specimens in the three groups during the torsion test (mean ± S.D., N∙m)

Item	I group	AF group	PF group	t_I-AF_	t_I-PF_	t_AF-PF_
LR	5.8 ± 0.5	7.7 ± 0.6	7.8 ± 0.5	< 0.001	< 0.001	0.820
RR	5.6 ± 0.4	7.9 ± 0.6	7.6 ± 0.5	< 0.001	< 0.001	0.259

*LR* left rotation and *RR* right rotation

**Fig. 6:**
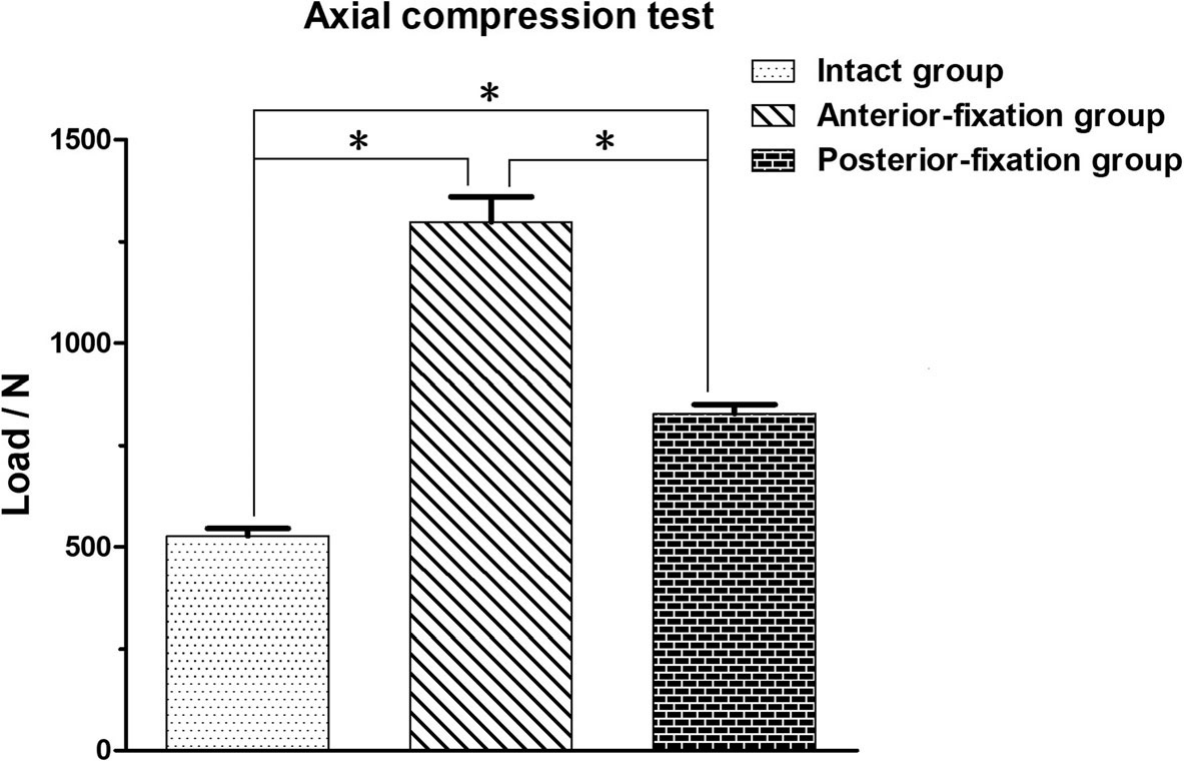
Load comparisons of the specimens in the three groups when the maximum loading displacement was achieved during the axial compression test. *P < 0.05 indicates that the difference was statistically significant

Under the maximum loading displacement (5 mm), the required load for the specimens in the AF group was significantly greater (*P* < 0.05) than that for the specimens in the PF group and I group in the axial compression direction; the load required for the specimens in the PF group was significantly greater than that in the I group (*P* < 0.05). The detailed information is shown in Fig. 6.

## Discussion

The lumbar spine is an important component that transmits the upper body weight to the lower limbs. The lumbar spine has a high incidence of diseases such as spondylolisthesis, fractures and disc degeneration due to a large range of motion and stress concentration [17–20]. Spinal cord decompression combined with dual screw-rod fixation through a single posterior approach has achieved satisfactory curative effects in the treatment of the above diseases and has been widely used in clinical practice [21]. However, diseases such as lumbosacral spinal tuberculosis and tumors often cause damage to the anterior vertebral columns and intervertebral discs, and it is difficult to thoroughly clear the lesions through the posterior approach alone because of the complex anatomy of the region. To solve the above deficiencies, some scholars have proposed an anteroposterior approach. Although this kind of operation has the advantages of sufficient clearing and solid fixation, the anteroposterior approach is still controversial because of the substantial trauma and high cost. Thus, a single anterior approach is favored by some surgeons, which has the advantage of clearing the lesions under direct vision. However, some previous studies have shown that the stability of anterior fixation was inferior to that of posterior fixation. Some anterior lumbosacral internal fixation systems have been designed to improve the strength of internal fixation, but these fixation systems cannot be applied to spinal tuberculosis patients who have irregular bony destruction due to defects of their own construction. To remedy the above deficiencies, we have developed a new surgical procedure, one-stage anterior debridement and cage implantation combined with anterior-lateral fixation by a dual screw-rod construct underneath the iliac vessel. After our long-term follow-up study, the therapeutic effect was deemed satisfactory, and no complications such as vascular rupture occurred. However, some surgeons question the fixation; therefore, we conducted an in vitro biomechanical study with human specimens to assuage these doubts.

The main purpose of this study was to observe the effects of anterior and posterior fixation on lumbosacral spine stability in the flexion, extension, lateral bending, rotation and axial compression directions. According to the results of the preliminary experiments and related literature reports, the maximum loading displacement of the bending test and axial compression test was set to 5 mm, while the maximum loading angle of the axial torsion test was set to 5 °. Prostheses yielding and vertebral fracturing were not observed throughout the whole experiment.

The results of the bending test showed that the loads of the specimens in the three groups increased as the displacement increased, which was consistent with the larger displacement caused by a larger load. The loads in the AF group and PF group were significantly higher than that of the I group in terms of unit-loading displacement in the flexion, extension and lateral bending directions, which may be related to the buffering effect of the intervertebral disc in the I group. Moreover, the results also showed that the two fixation methods could achieve better stability in the four movement directions. Under the maximum load displacement, the load of the AF group was significantly greater (*P* < 0.05) than that of the PF group in the flexion and left and right lateral bending directions, indicating that one-stage anterior debridement and cage implantation combined with anterior-lateral fixation by a dual screw-rod construct could achieve better stability than posterior dual screw-rod fixation in flexion and lateral bending. However, in the extension direction, the load required by the specimens of the PF group was significantly greater (*P* < 0.05) than that of the specimens of AF group. This finding suggested that the immediate stability of the AF group was slightly inferior to that of the PF group in extension. A possible reason for this phenomenon was that the titanium cage was located in the anterior column of the spine, and the cage provided only support and could not provide tension for the spine when it was not fused with adjacent vertebrae.

The axial torsion test showed that the load of the I group was smaller than those of the AF and PF groups under the maximum rotation angle (*P* < 0.05), while the loads of the latter two groups in the above direction were very similar (*P* > 0.05). These results showed that the disc acted as a buffer in the rotation of the spine and that both anterior fixation and posterior fixation could provide immediate stability for the lumbosacral spine in the rotation direction. The results of the axial compression test showed that the loads of the specimens in the AF and PF groups were significantly higher (*P* < 0.05) than that in the I group, which indicated that both fixation methods could meet the axial compression requirements of the lumbosacral spine. However, the load of the AF group was significantly higher than that of the PF group under the maximum loading displacement, indicating that the anterior fixation could better meet the axial compression requirements. The reason for this finding is that 80 to 90% of the pressure of the spine is mainly transmitted through the anterior column. The anterior fixation played a very good supporting role through the titanium cage, which enabled anterior fixation to exhibit better performance than posterior fixation under axial compression.

The limitations of this study must be acknowledged. First, although our results suggest that one-stage anterior debridement and cage implantation combined with anterior-lateral fixation by dual screw-rod could provide better immediate stability in the directions of flexion, extension, lateral bending, rotation and axial compression, the results should be cautiously interpreted because of the study’s low power. The use of a larger sample set should be considered in future studies. Second, the experiment only assessed the mechanical properties in vitro; the in vivo bio-safety shall be further studied.

## Conclusions

This study confirmed that one-stage anterior debridement and cage implantation combined with anterior-lateral fixation by a dual screw-rod construct could provide better immediate stability in the flexion, extension, lateral bending, rotation and axial compression directions. The stability provided by using this novel approach is better than that provided by posterior dual screw-rod fixation in flexion, lateral bending and axial compression. Although the stability of anterior fixation in extension was slightly worse than that of the posterior fixation, we believe that the stability of anterior fixation will gradually increase with the fusion of the titanium cage and surrounding bone, which may be no less than that of the posterior fixation.

## Abbreviations

AF: Anterior fixation
BMD: bone mineral density
CT: Computed tomography
EXT: Extension
FLX: Flexion
I: Intact
L: Lumbar
LLB: Left lateral bending
LR: Left rotation
PF: Posterior fixation
RLB: Right lateral bending
RR: Right rotation
S: Sacrum
S.D.: Standard deviation
